# Reversible Exfoliation‐Aggregation Dynamics in Ionic COFs for Tunable Interfacial Catalysis

**DOI:** 10.1002/advs.77122

**Published:** 2026-08-14

**Authors:** Jilu Yang, Xiaofei Zhang, Yue Li, Yuping Cao, Zhaowei Jia, Bo Li, Haitao Feng, Xiaoqiang Cui, Wenwu Qin

**Affiliations:** ^1^ State Key Laboratory of Applied Organic Chemistry Key Laboratory of Nonferrous Metal Chemistry and Resources Utilization of Gansu Province College of Chemistry and Chemical Engineering Lanzhou University Lanzhou People's Republic of China; ^2^ School of Materials Science and Engineering Key Laboratory of Automobile Materials of MOE Jilin University Changchun People's Republic of China; ^3^ State Key Laboratory of Supramolecular Structure and Materials College of Chemistry Jilin University Changchun People's Republic of China; ^4^ School of Chemistry and Chemical Engineering Guangxi Minzu University Nanning People's Republic of China; ^5^ Guangxi Key Laboratory of Al‐Driven Zero‐Carbon Technology Key Laboratory of New Low‐Carbon Green Chemical Technology Education Department of Guangxi Zhuang Autonomous Region School of Chemistry and Chemical Engineering Guangxi University Nanning People's Republic of China; ^6^ Key Laboratory of Comprehensive and Highly Efficient Utilization of Salt Lake Resources Qinghai Institute of Salt Lakes Chinese Academy of Sciences Xining People's Republic of China

**Keywords:** controllable‐exfoliation‐aggregation, covalent organic framework, dynamic combinatorial chemistry, photocatalytic hydrogen evolution, tailorable particle size

## Abstract

2D covalent organic frameworks (COFs) have emerged as promising functional materials for catalysis, adsorption, sensing, and energy storage. Their interfacial behavior and surface microenvironment play a decisive role in performance, yet these features are typically considered static or irreversible due to the lack of dynamic structure models. In this study, we report the construction of an ionic COF (iCOF), I‐4, designed through precise tuning of its skeleton and counterions, which uniquely exhibits reversable dynamic exfoliation and aggregation behavior in aqueous environments. This enables bidirectional control over particle size and phase states. Photocatalytic hydrogen evolution (PHE) studies reveal a strong correlation between solution concentration, particle size, and hydrogen production efficiency. Notably, I‐4 achieves a remarkable PHE rate of 190 mmol g^−1^ h^−1^ at 35°C. Moreover, the material can be fully precipitated and recovered from solution by simple iodide salt addition, enabling closed‐loop material recycling. Mechanistic analysis based on weak‐force interactions and soft‐hard acid‐base theory provides insight into the dynamic exfoliation process, highlighting the synergistic roles of counterion assembly and framework structure. These findings pave the way for the tailored synthesis of next‐generation dynamic COF materials with reversible interfacial adaptability, offering broad application potential in catalysis, ion transport, and molecular separation.

## Graphical abstract

A unique ionic covalent organic framework (I‐4) exhibits unprecedented concentration‐dependent dynamic behavior in water, undergoing reversible transitions between aggregation and exfoliation. This enables fully controllable, bidirectional modulation of its particle size and surface micro‐environment. Such dynamic transformation substantially reshapes the physicochemical properties of the material, thereby enabling precise regulation of its photocatalytic activity.

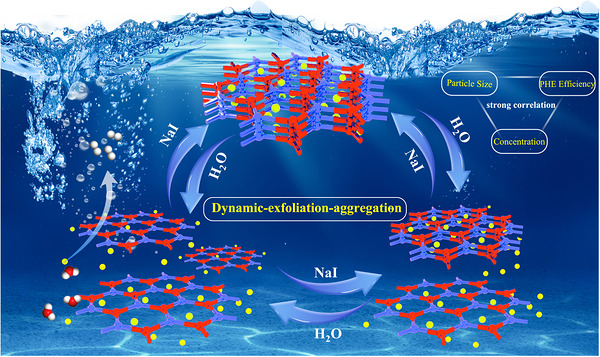

## 1 Introduction

**SCHEME 1:**
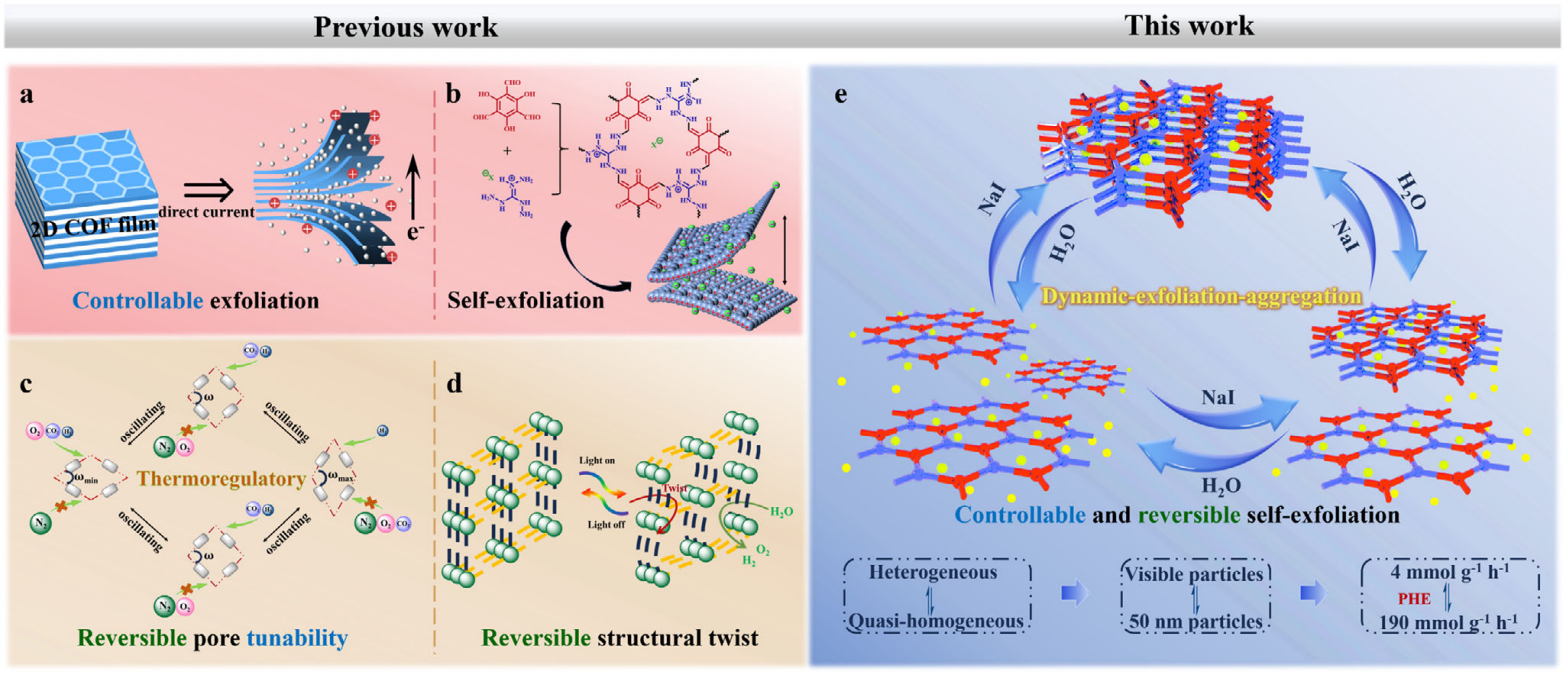
Exfoliating strategies for 2D organic materials (a, b), the application of dynamic structural control to porous materials (c, d) and Bidirectional control of particle size and phase state through dynamic‐exfoliation‐aggregation of materials (e). (a) High‐throughput electrochemical strategy to non‐destructively exfoliate polycrystalline 2D COF thin films into large nanosheets with long‐range crystallographic ordering. (b) The utilization of intrinsic ionic linker (guanidinium units) in conjunction with the rational design of COF can produce self‐exfoliating iCONs in the absence of external stimuli. (c) Temperature‐mediated dynamic modulation of skeleton oscillation enables sub‐0.2 Å precision molecular recognition in covalent organic frameworks. (d) Dynamic structural twist in metal‐organic frameworks enhances solar overall water splitting. (e) The dynamic‐exfoliation‐aggregation and PHE process of iCOF (I‐4) in water. The red and blue units represent the pyridinium ethyl cation and tri([1,1'‐biphenyl]‐4‐yl)amine (TBPA), respectively. The yellow balls inside and outside the framework represent fixed and free iodine ions on the skeleton, respectively.

Heterogeneous functional materials are among the most widely utilized platforms in catalysis, adsorption, and mass transfer, offering advantages such as high selectivity, extended lifetime, tunable structures, robust stability, environmental tolerance, and potential for recyclability [1, 2, 3, 4, 5, 6, 7]. These materials are also compatible with diverse energy input modalities, including light, electricity, and heat [8, 9, 10, 11, 12, 13, 14, 15, 16, 17]. However, a critical limitation persists: catalytic, adsorption, or stimulus‐responsive processes occur only at the external surface, leaving the internal reaction sites underutilized [18, 19]. This incomplete exploitation of active regions significantly hampers performance and constrains broader application. In contrast, homogeneous systems achieve more uniform site accessibility and utilization during reactions [20, 21]. Bridging the benefits of homogeneous systems into heterogeneous architectures is thus a key strategy for advancing next‐generation functional materials. To address the interfacial limitations of heterogeneous catalysts, various effective approaches have been proposed [22, 23, 24, 25]. For example, Zhang et al. pioneered the concept of single‐atom catalysis, demonstrating highly efficient atomic utilization via isolated Pt atoms anchored on iron oxide [26]. Zheng et al. reported a high‐loading Pd/TiO_2_ catalyst (1.5 wt%) synthesized through photochemical methods [27]. Another powerful strategy is the construction of quantum dots (QDs). Lu et al. used Mo_2_C–MoO_2_@RGO QDs of different sizes to systematically study how size modulates interfacial properties and catalytic activity [28]. Nanoscaling organic framework materials has also proven effective [29]. Feng and colleagues used surfactant templating to produce ultrathin 2D covalent organic frameworks (COF) nanosheets [30]. More recently, introducing repulsive charges to induce spontaneous self‐exfoliation has attracted significant interest [31]. Wang et al. developed a high‐throughput electrochemical method to exfoliate polycrystalline 2D COF films into large‐area nanosheets while preserving long‐range order (Scheme 1a) [32]. Similarly, Banerjee's team designed ionic COFs (iCOFs) with intrinsic positive charges, promoting exfoliation through electrostatic repulsion and significantly reducing exfoliation time under ultrasonic treatment (Scheme 1b) [33]. However, the tailored regulation of exfoliation in iCOFs has been rarely studied systematically.

Dynamic structural control has recently emerged as a transformative tool for structural and functional tuning in porous materials. It has enabled precise control over pore size distribution [34], catalytic kinetics [35], and could be further leveraged to modulate exfoliation‐recombination behavior in responsive materials (Scheme 1c,d). The integration of reversible non‐covalent interactions into 2D frameworks may offer a paradigm shift in the rational design of adaptive catalytic systems, unlocking new performance regimes and enabling reversibility and tunability at the interface level.

In this work, we report the design and synthesis of a series of iCOFs (I‐1 to I‐3 and X‐4 with counterions Cl^−^, Ac^−^, Br^−^, I^−^). By systematically varying the counterion species, framework architecture, catalyst concentration, and reaction temperature, we achieved multilevel dynamic regulation of interfacial behavior. Among these, the I‐4 variant featuring the largest pore size and a soft base I^−^ counterion, exhibited pronounced reversible exfoliation and aggregation behavior in water. At low concentrations, I‐4 dispersed uniformly and demonstrated significantly enhanced photocatalytic hydrogen evolution (PHE) activity, achieving a rate of 190.06 mmol g^−1^ h^−1^ at 35 °C. Upon addition of excess iodide, I‐4 readily precipitated, enabling closed‐loop material recovery (Scheme 1e). This study comprehensively investigates the dynamic exfoliation‐aggregation behavior of I‐4 across multiple scales‐from macroscopic catalytic performance to mesoscopic particle size and microscopic molecular assembly. Our findings not only provide mechanistic insight but also offer design principles for the precise and high‐throughput tailored synthesis of monodisperse 2D materials.

## 2 Results and Discussion

### 2.1 Synthesis and Characterizations I‐(1‐3) and X‐4

**FIGURE 1:**
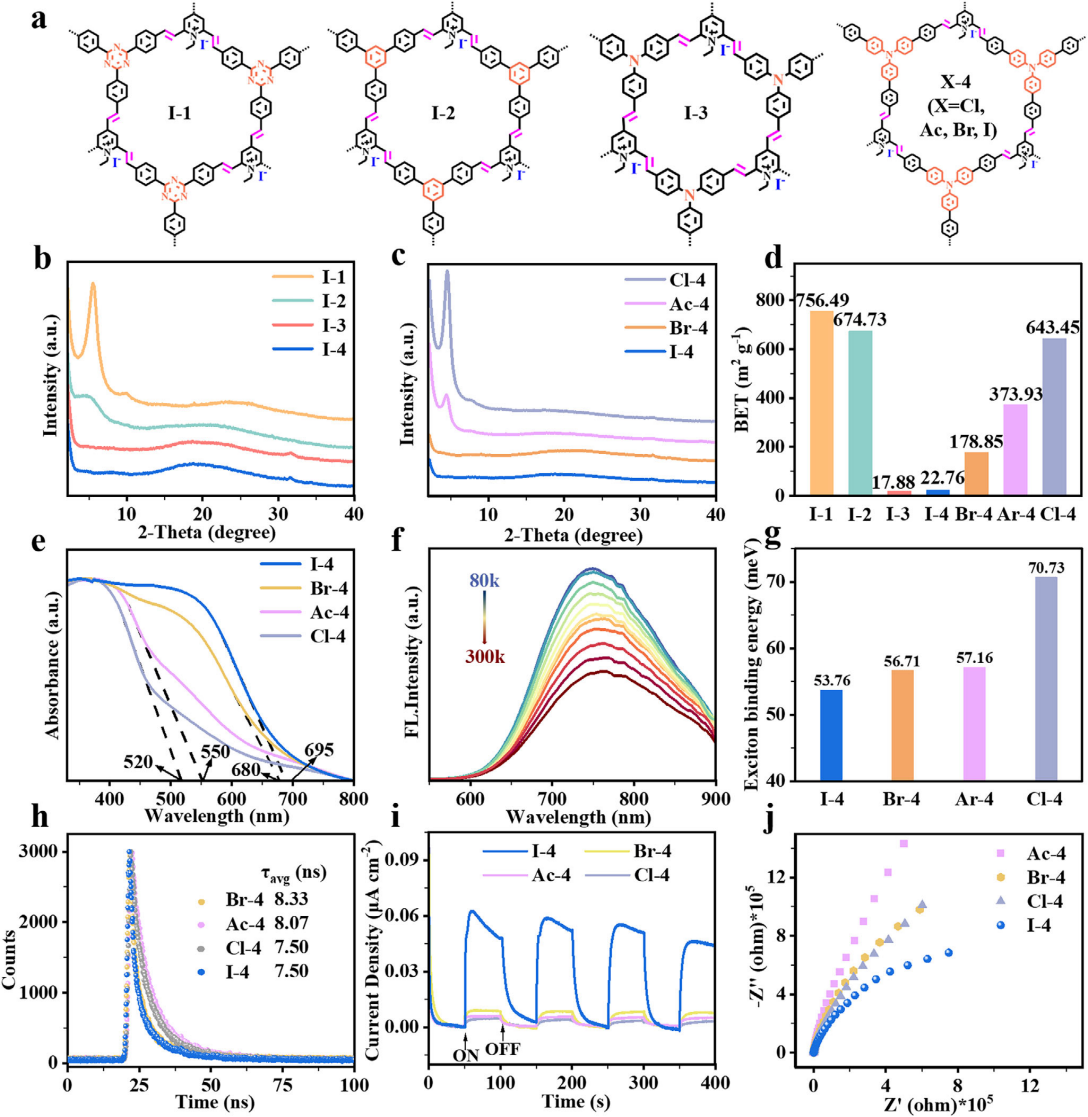
Structural characterization and Photoelectric properties of iCOFs. (a) Design and synthesis for iCOFs. PXRD patterns of (b) I‐(1‐4) and (c) X‐4. (d) BET of I‐(1‐3) and X‐4. (e) UV–vis diffuse reflectance spectroscopy of X‐4. (f) Temperature‐dependent PL spectra with excitation wavelength at 390 nm for I‐4 and the extracted exciton binding energies. (g) The exciton binding energy of X‐4. (h) Time‐resolved fluorescence (TRFS) spectra for X‐4. (i) Photocurrent measurements for X‐4. (j) EIS spectra of X‐4 under light illumination. (The impedance fitting results are shown in Table S1).

We synthesized I‐1 to I‐3 and X‐4 with counterions Cl^−^, Ac^−^, Br^−^, I^−^ from aldehyde monomers and ETMP‐X, which are linked by C═C (Figure 1a and Supporting Information) [36, 37]. Their structure and chemical composition had been demonstrated by X‐ray photoelectron spectroscopy (Figure S1), Ion chromatography (Figure S2) Fourier transform infrared (FTIR) spectroscopy, solid‐state ^13^C cross polarization magic angle spinning nuclear magnetic resonance spectroscopy (CP/MAS NMR), X‐ray spectroscopy (EDX) elemental maps and so forth. The FTIR peaks (Figures S3 and S4a) of iCOFs at 960 and 1629 cm^−1^, which could be attributed to the bending and stretching vibration of trans ─C═C, respectively. In the ^13^C NMR spectra (Figures S4b, S5, and S6), the characteristic chemical shift at 9.09–58.65 ppm was attributed to ─CH_3_ and ─CH_2_, and peak signals located at 109.35–165.49 ppm that originated from benzene and ─C═C, which provides evidence that the Knoevenagel conditioning was successfully implemented. The presence of Cl, O, Br, and I in the EDX elemental maps (Figures S7–S10) of Cl‐4, Ac‐4, Br‐4, and I‐4 further confirmed the successful synthesis of series X‐4. The powder X‐ray diffraction (PXRD) at room temperature can exhibit crystallinity of I‐(1‐3) and X‐4, with I‐1, I‐2, Cl‐4 and Ac‐4 displaying higher crystallinity, while I‐3, I‐4 and Br‐4 exhibit lower crystallinity (Figure 1b–d and Figures S11–S14). The thermogravimetric analysis (TGA) curve, as shown in Figure S15, indicates that X‐4 (where X═Cl^−^, Ac^−^, Br^−^, I^−^) exhibits favorable thermal stability. Upon heating to 800°C, the mass retention remains approximately 60%.

To clarify the band structure of X‐4, the solid‐state ultraviolet–visible (UV–Vis) spectroscopy of X‐4 is illustrated in Figure 1e, whereas I‐4 displays the most extensive visible light absorption range, which can extend up to 695 nm; the absorption edges of Cl‐4, Ac‐4, and Br‐4 are situated at 520, 550 and 680 nm, respectively. Accordingly, the optical bandgaps (E_g_) of Cl‐4, Ac‐4, Br‐4, and I‐4 are estimated to be 2.62, 2.49, 1.98, and 1.92 eV, respectively, based on the Tauc plot (Figure S16) [38, 39]. The flat band potential is established by the Mott‐Schottky (M‐S) plot (Figure S17), which indicates that X‐4 exhibit positive slope and therefore can be classified as n‐type semiconductor [40]. In comparison to E_fb_, the conduction band minimum (CBM) of n‐type semiconductors is typically observed to be slightly negative at 0.1 eV. The estimated CBM levels of Cl‐4, Ac‐4, Br‐4, and I‐4 are ‐0.51, ‐0.47, ‐0.84, and ‐1.12 eV (vs NHE), respectively. It is evident that their CBM values are considerably more negative than the potential for water splitting (0 V vs NHE), thereby providing sufficient driving force for water decomposition. In temperature‐dependent photoluminescence (PL) spectra (Figure 1f,g and Figure S18), I‐4 exhibits the smallest exciton binding energy. In the time‐resolved fluorescence (TRFS) spectra (Figure 1h and Figure S19) of different X‐4, I‐4 exhibits the shortest fluorescence lifetime, which could facilitate intramolecular charge transfer and diminish the likelihood of photo‐excited state electron deactivation [41, 42]. Meanwhile, the photocurrent–time curve (Figure 1i) illustrates that the photocurrent density of I‐4 is 7.5, 12, and 15 times that of Br‐4, Ac‐4, and Cl‐4, respectively, indicating that more photoinduced electrons can be generated. Electrochemical impedance spectroscopy (EIS) [43] (Figure 1j and Figure S20 and Table S1) revealed that I‐4 owns much smaller interfacial charge transfer resistance [44]. This is supported by the PL spectroscopy (Figure S21) and LSV curves (Figure S22).

### 2.2 The Impact of Dynamic‐Exfoliation on PHE

**FIGURE 2:**
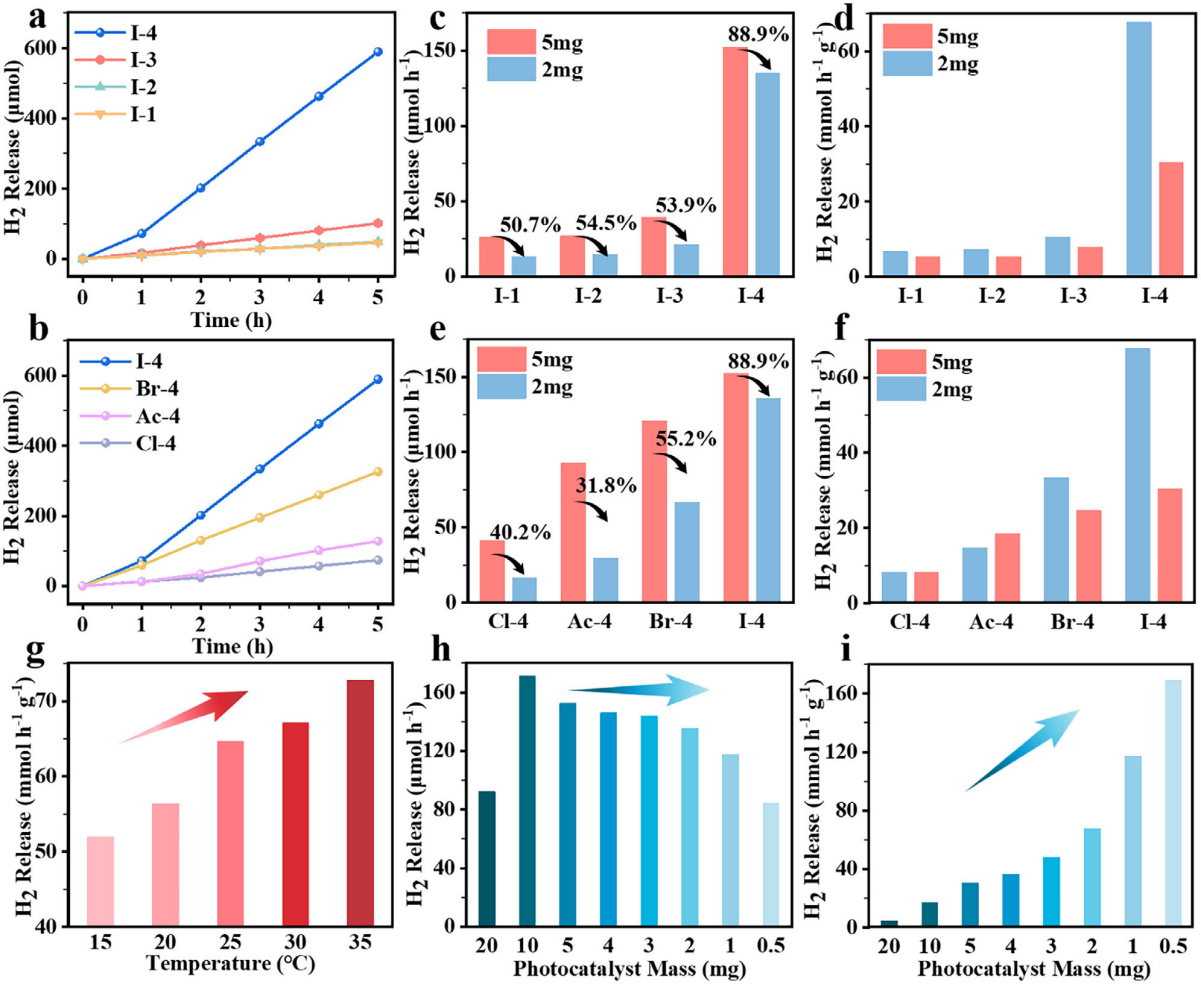
Hydrogen evolution performance of iCOFs. Time‐dependent photocatalytic H_2_ evolution of (a) I‐(1‐4) and (b) X‐4. The hydrogen production of (c) I‐(1‐4) and (e) X‐4 with 2 mg and 5 mg. The percentage represents the ratio of hydrogen evolution production from 2 mg iCOFs to 5 mg iCOFs. Mass‐normalized rates of H_2_ from (d) I‐(1‐4) and (f) X‐4 with 2 mg and 5 mg. (g) Temperature‐dependent photocatalytic H_2_ evolution of I‐4. (h) Quality‐dependent photocatalytic H_2_ evolution rates of I‐4. (i) Mass‐normalized rates of H_2_ from I‐4.

To shed light on the significance of dynamic‐exfoliation of I‐4 in PHE, iCOFs with varying frameworks and counter anions were subjected to photocatalytic experiments under visible light (*λ* > 420 nm). When 5 mg photocatalyst was added to 25 mL catalytic system, the highest photocatalytic hydrogen production (152.35 µmol h^−1^) was exhibited by I‐4, which uses I^−^ as counter anions, in conjunction with TBPA as building blocks (Figure 2a,b). Following this, we examined the influence of varying the amount of catalyst on the rate of hydrogen release (Figure 2c–f), the special catalytic activity exhibited by I‐4 caught our attention. In the absence of any other influencing factors, reducing the mass of photocatalyst to 2 mg resulted in hydrogen production of 135.45 µmol h^−1^, which equates to 88.9% with 5 mg. Subsequently, systematic study was conducted to investigate the effects of temperature (Figure 2g), catalyst concentration (Figure 2h,i), and Pt loading (different concentrations of photocatalysts) (Figure S23) on the hydrogen evolution efficiency of I‐4 in the catalytic system. It was observed that as temperature increased, the rate of hydrogen production demonstrated consistent upward trend. In addition, when the amount of catalyst is gradually reduced over a wide range, the hydrogen evolution rate of the entire system remains relatively stable, and there is no proportional decrease based on mass. Even if the amount of I‐4 is reduced to 0.5 mg (84.55 µmol h^−1^), the system can still maintain hydrogen production of 55.5%, compared to 5 mg. The optimal loading amount of Pt appear to be unaffected by alterations in the quality of I‐4. Surprisingly, after screening for temperature, concentration, and Pt loading, 0.5 mg of I‐4 exhibited remarkably high hydrogen evolution rate (190.06 mmol g^−1^ h^−1^) at 35°C (Figures S24–SS26), and demonstrated zero‐order kinetics. During the extended 15 h experiment, the hydrogen evolution performance of I‐4 remained unchanged, with no significant alterations observed in the FTIR and PXRD spectra (Figure S27). Meanwhile, TEM imaging, EDX elemental mapping, and AFM images clearly revealed the presence of Pt species on the catalyst surface (Figures S28 and S29).

To examine the influence of wavelength on the photocatalytic activity, we tested the hydrogen evolution activity of I‐4 at 420, 450, 500 and 550 nm, and calculated its apparent quantum yield (AQY). At 420 nm, the AQY of I‐4 is relatively high (Figure S30), reaching 2.2%, and then the comparable trend was observed between AQY and absorbance, which indicated that near‐infrared radiation can also effectively facilitate water splitting when I‐4 is utilized as a photocatalyst.

### 2.3 Dynamic‐Exfoliation‐Aggregation With I‐4

#### 2.3.1 Dynamic‐Exfoliation

**FIGURE 3:**
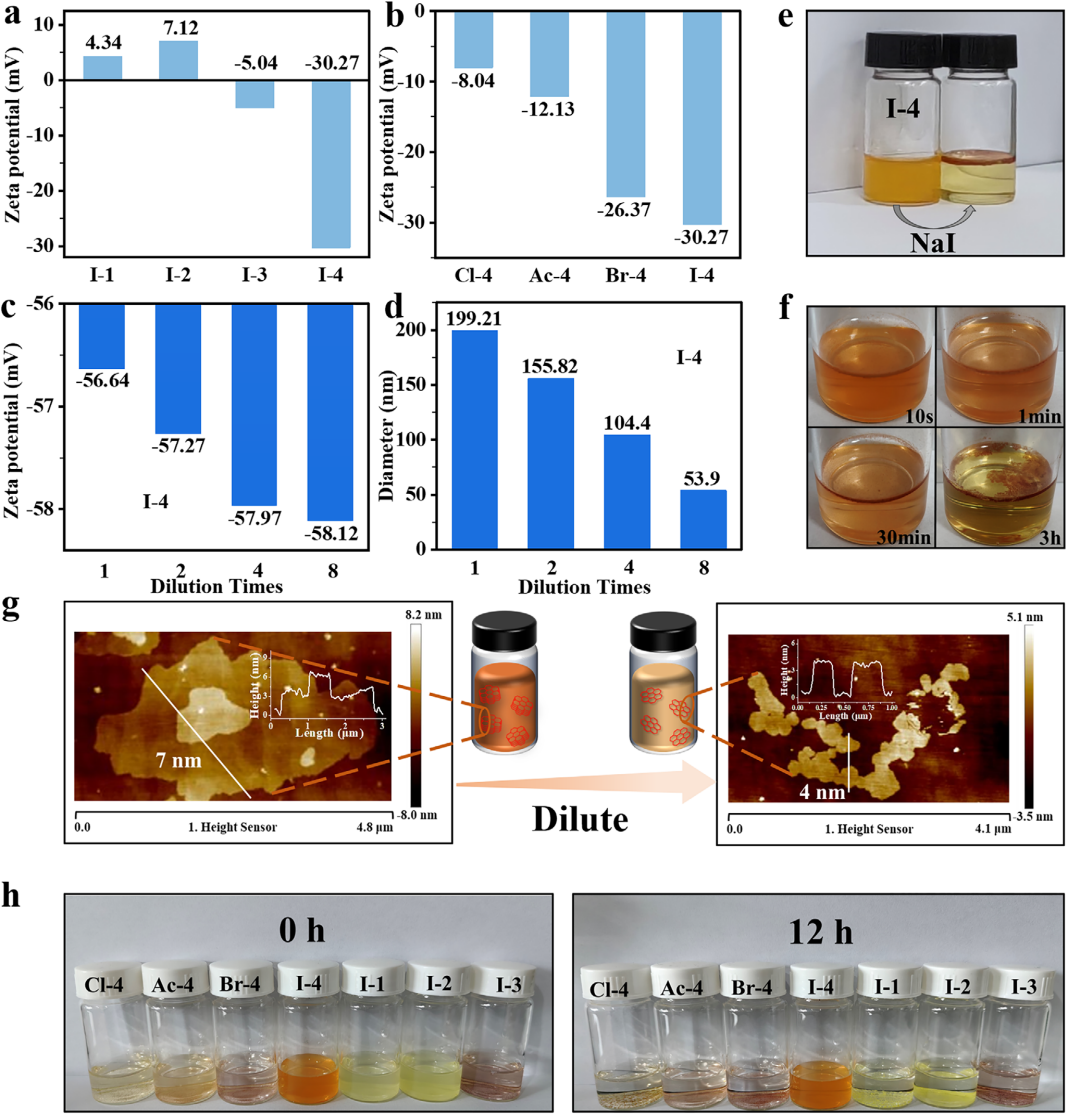
Dynamic‐exfoliating‐aggregating characteristics of iCOFs. Zeta potential of (a) I‐(1‐4) and (b) X‐4. (c) Zeta potential and (d) particle size of the initial I‐4 solution following gradient dilution. (e) Comparison photo after addition of NaI to I‐4 water system. (f) The aggregation situation of I‐4 colloid over time subsequent to the addition of NaI. (g) AFM images of I‐4 at high and low concentrations. The original solution (left) was diluted 8 times to obtain diluent (right). (h) Comparison photos of sedimentation of X‐4 and I‐(1‐3) at the same concentration at 0 h and 12 h.

In order to ascertain the impact of counterions and frameworks on the dynamic‐exfoliating degree of iCOFs, the zeta potentials of the initial I‐ (1–3) and X‐4 solutions were subjected to analysis (Figure 3a,b) [45, 46]. At the same concentration, the zeta potentials were observed to be 4.34 (I‐1), 7.12 (I‐2), ‐5.04 (I‐3), ‐8.04 (Cl‐4), ‐12.13 (Ac‐4), ‐26.37 (Br‐4) and ‐30.27 (I‐4), respectively. Among them, I‐4 showed the highest absolute value. Afterwards, the zeta potential of X‐4 were tested by continuously diluting the original solution (Figure 3c and Figure S31, detailed configuration details in Supporting Information). It was determined that only Br‐4 and I‐4 demonstrated distinctive attribute of progressively increasing zeta potential with declining concentration. For the purpose of gain more intuitive understanding of the exfoliating ability for different iCOFs, a series of particle size tests were conducted at varying concentrations (Figure 3d and Figures S32 and S33) [21, 47, 48]. However, it was found that Cl‐4, Ac‐4 and I‐(1‐3) could not be adequately stabilized during the testing process, resulting in rapid settling and inability to obtain stable particle size values. A modest decline is observed in Br‐4 as the concentration declines gradually. It is noteworthy that during the process of gradually decreasing concentration, I‐4 exhibits sustained, stable, and large span of particle size reduction, with notable correlation between the resulting particle size and the ratio of the material and water. This phenomenon has never been observed in organic framework materials, which provides compelling evidence for the highly distinctive dynamic‐exfoliating properties of the material and is able to explain the results of hydrogen production experiments very well.

The exfoliating behavior of iCOFs was also examined using atomic force microscopy (AFM), scanning electron microscopy (SEM), and high‐resolution transmission electron microscopy (HR TEM). In AFM images of I‐4 at varying concentrations (Figure 3g), it was observed that the nanosheets exhibited pronounced aggregation at elevated concentrations. However, after diluting the concentration to one‐eighth of the original, ultra‐thin nanosheet layers could be readily discerned. Despite the occurrence of aggregation at high concentrations, the thickness maintains highly uniform, with an average of approximately 3.5 nm. However, Ac‐4 still exhibited the phenomenon of multilayer stacking even under low concentration conditions (Figure S34). The same situation were also reflected in SEM and HR TEM images (Figures S35–S39) [49], where Cl‐4 and Ac‐4 were observed to be significantly thicker than Br‐4 and I‐4 at low concentrations, while X‐4 are all in aggregated state at high concentrations.

Comparative photographs of the sedimentation for X‐4 and I‐(1‐3) at the same concentration are also presented in Figure 3h and Figure S40. At 5 h, only Br‐4 and I‐4 exhibited incomplete settling. At 12 h, the I‐4 still did not settle, while the remaining iCOFs showed complete settlement. Even after four days of standing, I‐4 exhibited only minimal signs of settlement. Moreover, the contact angles of X‐4 with water were evaluated at time intervals of 0, 0.04, and 0.52 s (Figure S41), with the results demonstrating that X‐4 displayed excellent hydrophilicity.

#### 2.3.2 Dynamic‐Aggregation

In light of the aforementioned experimental outcomes, it can be speculated that the I‐4 displays unparalleled dynamic‐exfoliating‐aggregating characteristics and exhibits robust homogeneous phase features in aqueous environments [50, 51]. In other words, the product of the exposed cation skeleton sites of the I‐4 in water to the concentration of I^−^ remains approximately constant. With the increase of materials in the solution, or the concentration of I^−^ rises, the material will undergo aggregation to maintain constant product (Figure S42). This process bears resemblance to the dissolution of ionic compounds into homogeneous solutions in water. In experiments with different concentrations of particle size (Figure S32), the particle size of the material demonstrated declining trend with decreasing concentration, and consistently exhibited narrow particle size distribution, which well indicates the reconstruction process of the material particles and the reversible‐exfoliation of the material. In order to verify the dynamic aggregation process of the material, NaI was added to the I‐4 water system, thereby increasing the concentration of I^−^ (Figure 3e,f and Figure S43). Excitingly, when we added NaI to the I‐4 water system, flocs appeared and gradually increased over time. Even after 3 h, the I‐4 water system became almost clear. The experimental result provides compelling evidence that the delamination of I‐4 material is indeed reversible. Furthermore, photocatalytic hydrogen evolution tests conducted on the I‑4 recovered after closed‑loop recycling showed no significant loss in catalytic activity, further confirming that the dynamic exfoliation–aggregation process preserves the functional integrity of the material (Figure S44).

### 2.4 The Underlying Factors of Dynamic‐Exfoliation‐Aggregation

**FIGURE 4:**
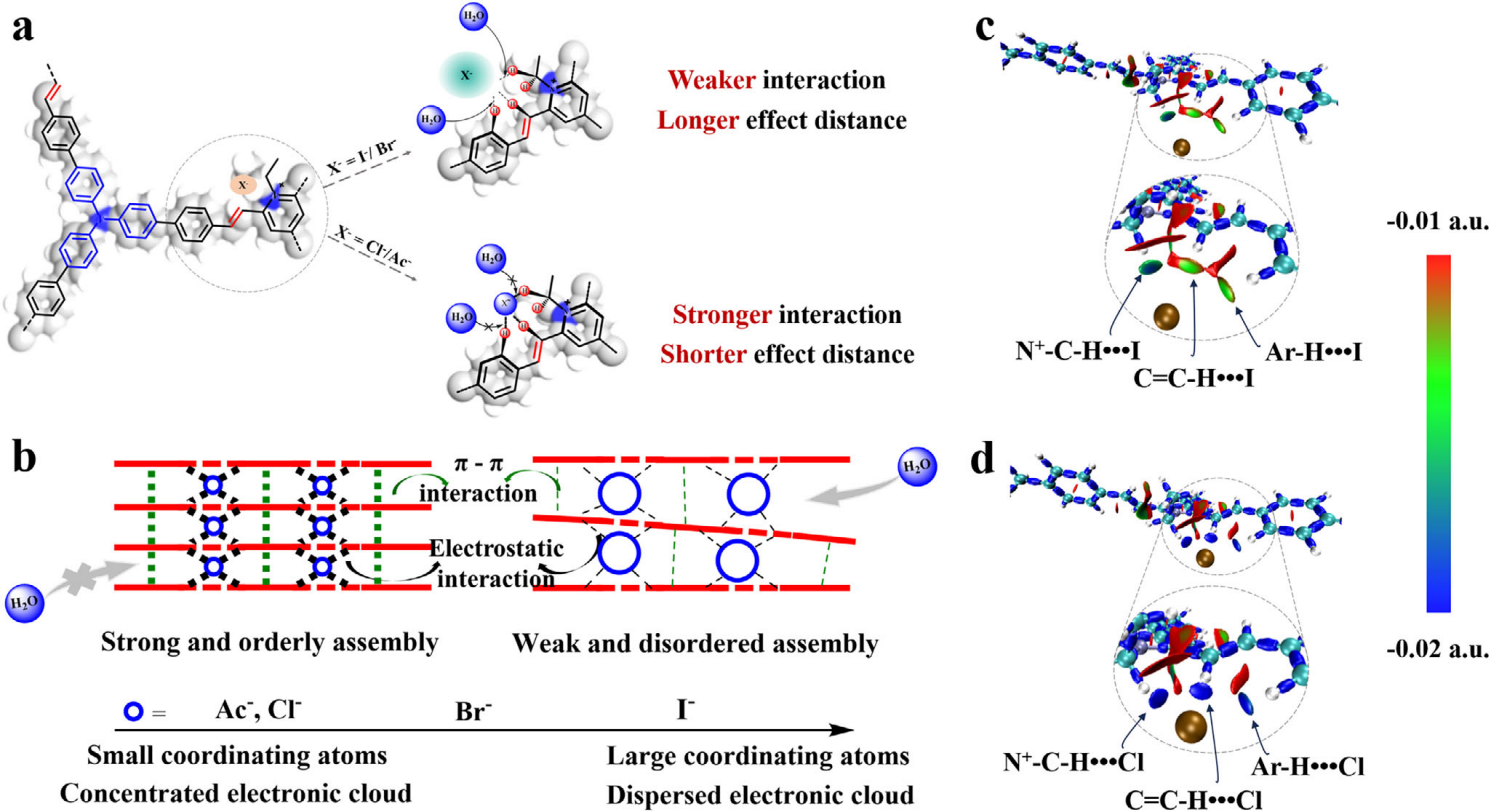
Mechanism explanation and theoretical calculation. (a) Schematic diagram of weak interaction of local fragment in X‐4. (b) Schematic diagram outlining the characteristics of counterions and their influence on the arrangement of the iCOFs. IRI analysis [52] of local fragment for (c) I‐4 and (d) Cl‐4, showing the non‐covalent interactions between the cationic skeleton and counter anion. The color scale is in the range of ‐0.02 < *ρ* < ‐0.01 a.u. Blue, green and red indicate regions of bond forming and notable interaction, moderate interaction, weak interaction and repulsion, respectively.

The exceptional catalytic properties and dynamic‐exfoliating characteristics of I‐4 have aroused our strong interest in render a comprehensive elucidation of the underlying chemical processes. Accordingly, we conducted DFT calculations on the iCOFs fragment structures of different counterions (Tables S2 and S3). It was discovered that in all X‐4 structures, the counter‐negative ions exhibit weak interactions with multiple hydrogen atoms on the skeleton (Figure 4a,c,d and Figure S45). However, the distance between different counter‐negative ions and hydrogen atoms on the skeleton varies significantly (Table S4). In circumstances where the negative charge (Cl^−^/Ac^−^‐hard base) is confined, the electrostatic behavior between the skeleton (H‐hard acid) and counterions is augmented, the interaction radius is diminished, and the assembly orderliness is elevated. Consequently, this results in high crystallinity of the materials and increased the kinetic energy barrier for exfoliating (Figure 4b left). Conversely, when the negative charge is relatively dispersed (Br^−^/I^−^‐soft base), the electrostatic interaction between the skeleton (H‐hard acid) and the counterions is weak, and the radius of action is longer. This, in turn, makes the assembly behavior of the material disordered and loose. The extremely low kinetic energy barrier allows the material to complete the dynamic‐exfoliation‐aggregation process in water (Figure 4b right).

The discrepancy in exfoliating capability between the I‐3 and I‐4 is another issue worthy of further investigation. we believe that this discrepancy is primarily attributable to alterations in pore size [53]. The pore size of I‐3 is smaller than I‐4 (Figure 1a), which results in greater occupation of space by I^−^. The narrow space also impedes the entry of water molecules, whereas I‐4 exhibits the opposite behavior. The sufficiently “soft” counter negative ions, large pore size, and low crystallinity afford I‐4 markedly advantageous exfoliation thermodynamics and kinetics, which collectively give rise to the material's distinctive dynamic‐exfoliating characteristics. The experimental evidence indicates that I‐4 exhibits the highest hydrophilicity, dispersion stability, and fastest dispersion speed among the materials subjected to the same pre‐treatment. Furthermore, the enhanced degree of dynamic‐exfoliation markedly elevates the utilization rate of effective active sites in I‐4, thereby conferring an ultra‐high photocatalytic hydrogen evolution efficiency to I‐4.

## 3 Conclusion

In this work, we report a series of ionic covalent organic frameworks, among which I‐4 exhibits excellent hydrophilicity and dispersion stability, as well as unprecedented dynamic‐exfoliating‐aggregating properties and quasi‐homogeneous characteristics. The distinctive characteristics of I‐4 were elucidated through particle size, zeta potential, and comparative experiments. The reasons for the exfoliating abilities of I‐(1‐3) and X‐4 were comprehensively and deeply explored through the hard‐soft‐acid‐base theory. With these in mind, I‐4 with high degree of exfoliation, markedly enhances the utilization rate of effective active sites. Furthermore, its quasi‐homogeneous characteristics effectively address the inherent phase interface separation issue in heterogeneous catalysts, exhibiting ultra‐high photocatalytic hydrogen evolution performance of 190 mmol g^−1^ h^−1^. More importantly, an initial structure‐activity relationship has been established between material microstructure, mesoscopic morphology, macroscopic physical behavior, and catalytic properties through the study of the physical and chemical properties of materials comprising different skeleton structures, pore sizes, and types of counterions. These discoveries provide principled guidance for the rational regulation of material exfoliating ability and interface characteristics. In future work, we will further explore methods for the precise customization of material particle sizes and roll out their application to size‐dependent scenarios such as drug delivery and photothermal therapy.

## Author Contributions

**Jilu Yang**: conceptualization, investigation, Writing – original draft, Writing – review and editing, software, data curation, and methodology. **Xiaofei Zhang**: conceptualization, writing – original draft, writing – review and editing, methodology, software, and data curation. **Zhaowei Jia**: writing – original draft. **Wenwu Qin**: conceptualization, investigation, funding acquisition, writing – original draft, writing – review and editing, visualization, validation, and methodology. **Xiaoqiang Cui**: conceptualization, investigation, writing – original draft, and writing – review and editing. **Bo Li**: writing – original draft, writing – review and editing, software, and funding acquisition. **Haitao Feng**: funding acquisition and validation. **Yue Li**: investigation. **Yuping Cao**: writing – original draft.

## Conflicts of Interest

The authors declare no conflicts of interest.

## Supporting information




**Supporting File 1**: advs77122‐sup‐0001‐SuppMat.docx.

## Data Availability

The data that support the findings of this study are available from the corresponding author upon reasonable request.

## References

[1] E. Gross , J. H.‐C. Liu , F. D. Toste , and G. A. Somorjai , “Control of Selectivity in Heterogeneous Catalysis by Tuning Nanoparticle Properties and Reactor Residence Time,” Nature Chemistry 4 (2012): 947–952, 10.1038/nchem.14651. 23089871

[2] A. T. Bell , “The Impact of Nanoscience on Heterogeneous Catalysis,” Science 299 (2003): 1688–1691, 10.1126/science.10836712. 12637733

[3] J. Huang , F. Zhu , W. He , F. Zhang , W. Wang , and H. Li , “Periodic Mesoporous Organometallic Silicas with Unary or Binary Organometals inside the Channel Walls as Active and Reusable Catalysts in Aqueous Organic Reactions,” Journal of the American Chemical Society 132 (2010): 1492–1493, 10.1021/ja909596a3. 20070082

[4] W. Gong , D. Chu , H. Jiang , X. Chen , Y. Cui , and Y. Liu , “Permanent Porous Hydrogen‐Bonded Frameworks with Two Types of Brønsted acid Sites for Heterogeneous Asymmetric Catalysis,” Nature Communications 10 (2019): 600, 10.1038/s41467-019-08416-64. PMC6363736 30723208

[5] S. Karakalos , Y. Xu , F. Cheenicode Kabeer , et al., “Noncovalent Bonding Controls Selectivity in Heterogeneous Catalysis: Coupling Reactions on Gold,” Journal of the American Chemical Society 138 (2016): 15243–15250, 10.1021/jacs.6b094505. 27775885

[6] X. Fang , L. Yang , Z. Dai , et al., “Poly(ionic liquid)s for Photo‐Driven CO_2_ Cycloaddition: Electron Donor–Acceptor Segments Matter,” Advanced Science 10 (2023): 2206687, 10.1002/advs.2022066876. 36642842 PMC10015876

[7] T. Wang , M. Li , J. Huang , R. Yang , and C. Li , “Benzotriazole‐Based Ionic COFs Embedded in Biologic Aerogels for Stable Photocatalytic Seawater Splitting,” Journal of Materials Chemistry A 14 (2026): 23112–23121, 10.1039/d6ta01764a7.

[8] D. Teschner , G. Novell‐Leruth , R. Farra , et al., “In Situ Surface Coverage Analysis of RuO_2_ ^−^ Catalysed HCl Oxidation Reveals the Entropic Origin of Compensation in Heterogeneous Catalysis,” Nature Chemistry 4 (2012): 739–745, 10.1038/nchem.14118. 22914195

[9] B. Han , Y. Jin , B. Chen , et al., “Maximizing Electroactive Sites in a Three‐Dimensional Covalent Organic Framework for Significantly Improved Carbon Dioxide Reduction Electrocatalysis,” Angewandte Chemie International Edition 61 (2022): 202114244, 10.1002/anie.2021142449. 34716743

[10] Z. Zhang , Z. Zhang , C. Chen , et al., “Single‐Atom Platinum with Asymmetric Coordination Environment on Fully Conjugated Covalent Organic Framework for Efficient Electrocatalysis,” Nature Communications 15 (2024): 2556, 10.1038/s41467-024-46872-x10. PMC10960042 38519497

[11] L. Bai , C.‐S. Hsu , D. T. L. Alexander , H. M. Chen , and X. Hu , “Double‐Atom Catalysts as a Molecular Platform for Heterogeneous Oxygen Evolution Electrocatalysis,” Nature Energy 6 (2021): 1054–1066, 10.1038/s41560-021-00925-311.

[12] X. Zhao , P. Pachfule , S. Li , et al., “Macro/Microporous Covalent Organic Frameworks for Efficient Electrocatalysis,” Journal of the American Chemical Society 141 (2019): 6623–6630, 10.1021/jacs.9b0122612. 30916950

[13] H. Li , C. R. Bowen , H. Dan , and Y. Yang , “Pyroelectricity Induced by Schottky Interface above the Curie Temperature of Bulk Materials,” Joule 8 (2024): 401–415, https://www.sciencedirect.com/science/article/pii/S254243512300498113.

[14] T. Shi , H. Wang , L. Li , et al., “Enhanced Photostability in Protonated Covalent Organic Frameworks for Singlet Oxygen Generation,” Matter 5 (2022): 1004–1015, https://www.sciencedirect.com/science/article/pii/S259023852200003014.

[15] B. Xie , D. Hu , P. Kumar , V. V. Ordomsky , A. Y. Khodakov , and R. Amal , “Heterogeneous Catalysis via Light‐Heat Dual Activation: A Path to the Breakthrough in C1 Chemistry,” Joule 8 (2024): 312–333, https://www.sciencedirect.com/science/article/pii/S254243512300531715.

[16] G. B. Park , T. N. Kitsopoulos , D. Borodin , et al., “The Kinetics of Elementary Thermal Reactions in Heterogeneous Catalysis,” Nature Reviews Chemistry 3 (2019): 723–732, 10.1038/s41570-019-0138-716.

[17] M. Ko , Y. Kim , J. Woo , et al., “Direct Propylene Epoxidation with Oxygen Using a Photo‐Electro‐Heterogeneous Catalytic System,” Nature Catalysis 5 (2022): 37–44, 10.1038/s41929-021-00724-917.

[18] Q. Zhu , C. J. Murphy , and L. R. Baker , “Opportunities for Electrocatalytic CO_2_ Reduction Enabled by Surface Ligands,” Journal of the American Chemical Society 144 (2022): 2829–2840, 10.1021/jacs.1c1150018. 35137579

[19] H. Zou , S. Shu , W. Yang , et al., “Steering Acidic Oxygen Reduction Selectivity of Single‐Atom Catalysts through the Second Sphere Effect,” Nature Communications 15 (2024): 10818, 10.1038/s41467-024-55116-x19. PMC11686109 39737986

[20] W. Wang , S. Wang , X. Ma , and J. Gong , “Recent Advances in Catalytic Hydrogenation of Carbon Dioxide,” Chemical Society Reviews 40 (2011): 3703–3727, 10.1039/C1CS15008A20. 21505692

[21] X. Wang , Z. Fu , L. Zheng , et al., “Covalent Organic Framework Nanosheets Embedding Single Cobalt Sites for Photocatalytic Reduction of Carbon Dioxide,” Chemistry of Materials 32 (2020): 9107–9114, 10.1021/acs.chemmater.0c0164221.

[22] X. Hai , Y. Zheng , Q. Yu , et al., “Geminal‐Atom Catalysis for Cross‐Coupling,” Nature 622 (2023): 754–760, 10.1038/s41586-023-06529-z22. 37730999

[23] X.‐B. Li , Z.‐J. Li , Y.‐J. Gao , et al., “Mechanistic Insights into the Interface‐Directed Transformation of Thiols into Disulfides and Molecular Hydrogen by Visible‐Light Irradiation of Quantum Dots,” Angewandte Chemie International Edition 53 (2014): 2085–2089, 10.1002/anie.20131024923. 24470069

[24] L. Zhang , Z. Yang , S. Feng , et al., “Metal Telluride Nanosheets by Scalable Solid Lithiation and Exfoliation,” Nature 628 (2024): 313–319, 10.1038/s41586-024-07209-224. 38570689

[25] J. Yang , Y. Zhang , Y. Ge , et al., “Interlayer Engineering of Layered Materials for Efficient Ion Separation and Storage,” Advanced Materials 36 (2024): 2311141, 10.1002/adma.20231114125. 38306408

[26] B. Qiao , A. Wang , X. Yang , et al., “Single‐Atom Catalysis of CO Oxidation Using Pt1/FeO_x_ ,” Nature Chemistry 3 (2011): 634–641, 10.1038/nchem.109526. 21778984

[27] P. Liu , Y. Zhao , R. Qin , et al., “Photochemical Route for Synthesizing Atomically Dispersed Palladium Catalysts,” Science 352 (2016): 797–800, 10.1126/science.aaf525127. 27174982

[28] Y. Wan , Z. Wang , J. Li , and R. Lv , “Mo_2_C‐MoO_2_ Heterostructure Quantum Dots for Enhanced Electrocatalytic Nitrogen Reduction to Ammonia,” ACS Nano 16 (2022): 643–654, 10.1021/acsnano.1c0797328. 34964347

[29] W. Zhao , L. Luo , M. Cong , et al., “Nanoscale Covalent Organic Frameworks for Enhanced Photocatalytic Hydrogen Production,” Nature Communications 15 (2024): 6482, 10.1038/s41467-024-50839-329. PMC11294449 39090140

[30] K. Liu , H. Qi , R. Dong , et al., “On‐Water Surface Synthesis of Crystalline, Few‐Layer Two‐Dimensional Polymers Assisted by Surfactant Monolayers,” Nature Chemistry 11 (2019): 994–1000, 10.1038/s41557-019-0327-530. 31548668

[31] K. Koner , H. S. Sasmal , D. Shetty , and R. Banerjee , “Thickness‐Driven Synthesis and Applications of Covalent Organic Framework Nanosheets,” Angewandte Chemie International Edition 63 (2024): 202406418, 10.1002/anie.20240641831. 38726702

[32] T. Ju , X. Shi , Z. Si , et al., “High‐Throughput Electrochemical Delamination of Two‐Dimensional Covalent Organic Frameworks,” Journal of the American Chemical Society 147 (2025): 23809–23818, 10.1021/jacs.5c0602632. 40560811

[33] S. Mitra , S. Kandambeth , B. P. Biswal , et al., “Self‐Exfoliated Guanidinium‐Based Ionic Covalent Organic Nanosheets (iCONs),” Journal of the American Chemical Society 138 (2016): 2823–2828, 10.1021/jacs.5b1353333. 26866697

[34] Y. Hu , B. Sengupta , H. Long , et al., “Molecular Recognition with Resolution below 0.2 Angstroms through Thermoregulatory Oscillations in Covalent Organic Frameworks,” Science 384 (2024): 1441–1447, 10.1126/science.adj879134. 38935724

[35] K. Sun , Y. Huang , F. Sun , et al., “Dynamic Structural Twist in Metal–organic Frameworks Enhances Solar Overall Water Splitting,” Nature Chemistry 16 (2024): 1638–1646, 10.1038/s41557-024-01599-635. 39134777

[36] F. Meng , S. Bi , Z. Sun , et al., “Synthesis of Ionic Vinylene‐Linked Covalent Organic Frameworks through Quaternization‐Activated Knoevenagel Condensation,” Angewandte Chemie International Edition 60 (2021): 13614–13620, 10.1002/anie.20210437536. 33844881

[37] J. Yang , X. Zhang , W. Si , et al., “Alkene‐Bridged Ionic Covalent Organic Nanosheets (iCONs) Based on D‐π‐A for Photocatalytic Hydrogen Evolution,” ACS Catalysis 14 (2024): 2022–2030, 10.1021/acscatal.3c0303437.

[38] S. Xu , H. Sun , M. Addicoat , et al., “Thiophene‐Bridged Donor–Acceptor sp^2^‐Carbon‐Linked 2D Conjugated Polymers as Photocathodes for Water Reduction,” Advanced Materials 33 (2021): 2006274, 10.1002/adma.20200627438. 33191503 PMC11468691

[39] K. Umezawa , Y. Nakamura , H. Makino , D. Citterio , and K. Suzuki , “Bright, Color‐Tunable Fluorescent Dyes in the Visible−Near‐Infrared Region,” Journal of the American Chemical Society 130 (2008): 1550–1551, 10.1021/ja077756j39. 18193873

[40] S. Wei , F. Zhang , W. Zhang , et al., “Semiconducting 2D Triazine‐Cored Covalent Organic Frameworks with Unsubstituted Olefin Linkages,” Journal of the American Chemical Society 141 (2019): 14272–14279, 10.1021/jacs.9b0621940. 31430139

[41] Y. Chen , R. Liu , Y. Guo , et al., “Hierarchical Assembly of Donor–acceptor Covalent Organic Frameworks for Photosynthesis of Hydrogen Peroxide from Water and Air,” Nature Synthesis 3 (2024): 998–1010, 10.1038/s44160-024-00542-441.

[42] Z. Zhang and Y. Xu , “Hydrothermal Synthesis of Highly Crystalline Zwitterionic Vinylene‐Linked Covalent Organic Frameworks with Exceptional Photocatalytic Properties,” Journal of the American Chemical Society 145 (2023): 25222–25232, 10.1021/jacs.3c0822042. 37856866

[43] M.‐L. Xu , M. Lu , G.‐Y. Qin , et al., “Piezo‐Photocatalytic Synergy in BiFeO_3_@COF Z‐Scheme Heterostructures for High‐Efficiency Overall Water Splitting,” Angewandte Chemie International Edition 61 (2022): 202210700, 10.1002/anie.20221070043. 36098495

[44] Y. Qian , Y. Han , X. Zhang , G. Yang , G. Zhang , and H.‐L. Jiang , “Computation‐Based Regulation of Excitonic Effects in Donor‐Acceptor Covalent Organic Frameworks for Enhanced Photocatalysis,” Nature Communications 14 (2023): 3083, 10.1038/s41467-023-38884-w44. PMC10227069 37248231

[45] T. Lin , I.‐W. Chen , F. Liu , et al., “Nitrogen‐Doped Mesoporous Carbon of Extraordinary Capacitance for Electrochemical Energy Storage,” Science 350 (2015): 1508–1513, 10.1126/science.aab379845. 26680194

[46] C. Wang , Y. Lu , Z. Wang , et al., “Salt‐Assisted Construction of Hydrophilic Carbon Nitride Photocatalysts with Abundant Water Molecular Adsorption Sites for Efficient Hydrogen Production,” Applied Catalysis B: Environment and Energy 350 (2024): 123902, https://www.sciencedirect.com/science/article/pii/S092633732400216946.

[47] F. Yang , J. Guo , C. Han , et al., “Turing Covalent Organic Framework Membranes via Heterogeneous Nucleation Synthesis for Organic Solvent Nanofiltration,” Science Advances 10 (2024): adr9260, 10.1126/sciadv.adr926047. PMC11633759 39661688

[48] B. J. Willner , C. M. Aitchison , F. Podjaski , et al., “Correlation between the Molecular Properties of Semiconducting Polymers of Intrinsic Microporosity and Their Photocatalytic Hydrogen Production,” Journal of the American Chemical Society 146 (2024): 30813–30823, 10.1021/jacs.4c0854948. 39475215 PMC11565637

[49] I. Krivtsov , D. Mitoraj , C. Adler , et al., “Water‐Soluble Polymeric Carbon Nitride Colloidal Nanoparticles for Highly Selective Quasi‐Homogeneous Photocatalysis,” Angewandte Chemie International Edition 59 (2020): 487–495, 10.1002/anie.20191333149. 31659848 PMC6973021

[50] H. Rao , L. C. Schmidt , J. Bonin , and M. Robert , “Visible‐Light‐Driven Methane Formation from CO_2_ with a Molecular Iron Catalyst,” Nature 548 (2017): 74–77, 10.1038/nature2301650. 28723895

[51] H. Takeda , K. Ohashi , A. Sekine , and O. Ishitani , “Photocatalytic CO_2_ Reduction Using Cu(I) Photosensitizers with a Fe(II) Catalyst,” Journal of the American Chemical Society 138 (2016): 4354–4357, 10.1021/jacs.6b0197051. 27015323

[52] T. Lu and F. Chen , “Multiwfn: A Multifunctional Wavefunction Analyzer,” Journal of Computational Chemistry 33 (2012): 580–592, 10.1002/jcc.2288552. 22162017

[53] R. Cao , R. B. Rossdeutcher , Y. Zhong , et al., “Aromatic Pentaamide Macrocycles Bind Anions with High Affinity for Transport across Biomembranes,” Nature Chemistry 15 (2023): 1559–1568, 10.1038/s41557-023-01315-w53. 37814114

