# Supplementary material for: Reversible Exfoliation‐Aggregation Dynamics in Ionic COFs for Tunable Interfacial Catalysis
Source: Adv Sci (Weinh). 2026 Aug 14:e77122. Online ahead of print. doi: 10.1002/advs.77122 (PMC13474177; doi:10.1002/advs.77122)
Supplement: Supplementary file 1 [file ADVS-9999-e77122-s001.docx]

**Supplementary Information**

**Reversible Exfoliation-Aggregation Dynamics in Ionic COFs for Tunable Interfacial Catalysis**

Jilu Yang^a,b^, Xiaofei Zhang^a,c^*, Yue Li^a^, Yuping Cao^d^, Zhaowei Jia^e^, Bo Li^f^, Haitao Feng^f^, Xiaoqiang Cui^b^*, Wenwu Qin^a^*

^a^ State Key Laboratory of Applied Organic Chemistry, Key Laboratory of Nonferrous Metal Chemistry and Resources Utilization of Gansu Province, College of Chemistry and Chemical Engineering, Lanzhou University, Lanzhou 730000, People’s s Republic of China.

^b^ School of Materials Science and Engineering, Key Laboratory of Automobile Materials of MOE, Jilin University, Changchun 130012, People’s Republic of China.

^c^ State Key Laboratory of Supramolecular Structure and Materials, College of Chemistry, Jilin University; Changchun 130012, People’s Republic of China.

^d^ School of Chemistry and Chemical Engineering, Guangxi Minzu University; Nanning 530006, P People’s Republic of China.

^e^ Guangxi Key Laboratory of Al-Driven Zero-Carbon Technology, Key Laboratory of New Low-carbon Green Chemical Technology, Education Department of Guangxi Zhuang Autonomous Region, School of Chemistry and Chemical Engineering, Guangxi University; Nanning 530004, People’s Republic of China.

^f^ Key Laboratory of Comprehensive and Highly Efficient Utilization of Salt Lake Resources, Qinghai Institute of Salt Lakes, Chinese Academy of Sciences; Xining 810008, People’s Republic of China.

***Corresponding Author:** Xiaofei Zhang; Xiaoqiang Cui; Wenwu Qin

**E-mail:** zhangxf24@mails.jlu.edu.cn; xqcui@jlu.edu.cn; qinww@lzu.edu.cn

**Materials and Method**

**Reagent:**

tris (4-bromophenyl) amine, 4-formylphenylboronic acid, 2,4, 6-trimethylpyridine and 5'-(4-Formylphenyl)-[1,1',3',1''-terphenyl]-4,4''-dicarbaldehyde **(TAPB)** were purchased in bidepharm (Shanghai China). Bromoethane, L-Ascorbic acid, Pd(PPh_3_)_4_, 2 M dimethylamine in anhydrous THF solution, N, N-dimethyl formamide **(DMF)**, ortho-dichlorobenzene **(o-DCB)**, n-butanol **(n-BuOH)** and N-butyl lithium were purchased in Energy Chemical (shanghai China) with super-dry grade. o-dichlorobenzene was obtained from HEOWNC (Tianjin China). Tetrahydrofuran **(THF)** was obtained from Tianjin Fuyu Fine Chemical Co., Ltd. (Tianjin China). Acetone was purchased in Xilong Science (Sichuan China). L-Ascorbic Acid Sodium Salt Sodium was obtained from Shanghai Aladdin Bio-Chem Technology Co., L TD. 4,4',4''-Nitrilotribenzaldehyde **(TPA)**, AgCl and CH_3_COOAg was purchased in Energy Chemical (shanghai China) and further purified. C_2_H_5_ONa was obtained from J&K. petroleum ether, K_2_CO_3_, Methanol **(MeOH)**, Dichloromethane **(DCM)**, N, N-dimethylacetamide **(DMAC)**, and Potassium Bromide **(KBr)** were obtained from DAMO CHEMICAL REAGENT FACTORY (Tianjin China). ethyl acetate and Acetonitrile were prepared in CHROH CHEMICALS. hydrochloric acid **(HCl)** was obtained from Chengdu Kelong Chemical Co., Ltd. (Chengdu China). 1,4-dioxane was purchased ZHONGTAI CHEMICAL.

**Equipment:**

Fourier Transform Infrared (FT-IR) spectroscopy

FT-IR spectroscopic measurements were recorded on a Nicolet is5 instrument (manufactured by Thermo Fisher Scientific) with a spectral range of 4000-800 cm^-1^ using the KBr disk method.

X-ray photoelectron spectroscopy (XPS)

The samples were pressed onto indium foil and the spectra were acquired by Shimadzu Axis Supra Japan X-ray photoelectron spectrometer. All of the XPS spectra presented in the main paper, and all subsequent data analysis were processed using the software XPS Peak Fit 4.1 and performed following subtraction of a Shirley background from the region of interest. The spectra were referenced to the adventitious C_1s_ peak at 284.800 eV.

Solution nuclear magnetic resonance (NMR) spectroscopy

^1^H spectra of the monomers ETMP-Br, ETMP-I, ETMP-Cl, ETMP-Ac, TFPT, TAPB, TPA, TBPA were recorded on a nuclear magnetic 400 M ^1^H NMR spectrum (JNM-ECS), where chemical shifts (δ in ppm) were determined with a residual proton of the solvent as standard.

Solid-State NMR (SSNMR) Experimental

The Solid-state ^13^C CP-MAS NMR spectra were recorded on Bruker AVANCE III 400 WB spectrometer. The powder samples were placed in a pencil-type zirconia rotor of 4.0 mm o.d.

Powder X-ray diffraction (PXRD)

PXRD patterns were measured on a Rigaku Ultima IV Japan with a Cu-Kα_1_-radiation (λ_1_ =1.540593 Å, λ_2_ =1.544427 Å). Samples were mounted on a flat sample plate. Patterns were collected in the 2^°^ < 2θ < 40^°^ range with a step size of 0.02^°^and scan speed of 5^°^ min^-1^.

Nitrogen adsorption-desorption isotherm measurements

The measurements were measured using a TriStar3020/ASAP2020M (USA) volumetric instrument with extra-high pure gases. Before the gas adsorption measurements, the as-prepared samples (100 mg) were dried under dynamic vacuum at 80 °C for 12 h. The Brunauer-Emmet-Teller (BET) was carried out to determine the total specific surface areas for the N_2_ isotherms at 77 K.

By using the non-local density functional theory (NLDFT) model, the pore size distributions were derived from the sorption curves.

Thermogravimetric analyses (TGA)

TGA analysis of samples was run on a Linseis Corporation PT 1600 thermal gravimetric analyzer with samples held in an aluminum oxide pan under nitrogen flow (60 mL/min). A 10 K·min^-1^ ramp rate was used from 25 to 800 °C.

Atomic Force Microscopy (AFM)

Mount the sample on the sample stage of atomic force microscopy (BRUKER NanoScope 6) and obtain the morphology of the sample. Atomic force microscopy measurements are used SCM-PIT-V2 as model (T = 2.8 μm, L = 225 μm, W = 35 μm, F_0_ = 75 kHz, k = 3 N/m).

Scanning Electron Microscopy (SEM)

Take a small amount of suspended liquid from the sample and drop it onto the silicon wafer and stick it onto the conductive adhesive. Use the Quorum SC7620 sputtering coating instrument to spray gold for 45 s, with a gold spray rate of 10 mA; Subsequently, the morphology of the sample was captured using a ZEISS Sigma 300 scanning electron microscope, with an accelerating voltage of 3 kV and a detector of SE2 secondary electron.

Transmission Electron Microscopy (TEM)

Take a portion of the sample and disperse it into an ethanol solution for ultrasound. Then, take a few drops of the dispersed liquid and add them dropwise to the copper mesh. After drying, the sample is subjected to vacuum testing on the machine, with Japanese JEOL JEM-F200 acceleration voltage of 200 kV.

Photoluminescence (PL) spectroscopy

PL spectroscopy were collected with a F-7000 spectrofluorometer at room temperature.

The time-resolved fluorescence decay spectrum were obtained on a FLS920 spectrofluorometer (Edinburgh Instruments) with an excitation wavelength of 300 nm of Cl/Ac-4 and 270 nm of Br/I-4, respectively. The average photoluminescence lifetime were estimated using Equation as follows:

τ = τ_1_𝑅_1_ + τ_2_𝑅_2_ + τ_3_𝑅_3_

Where τ is average decay time, τ_1_, τ_2_ and τ_3_ are decay times were fitted by triexponenital kinetics function, and R_1_, R_2_ and R_3_ are relative magnitudes.

Temperature-dependent PL spectra were collected in Fluorolog-QM-75-22 (HORIBA).

Contact Angle Measurements

The contact angle measurements were performed using Shanghai Xuanzhun SZ-CAMC32 Video Contact Angle Tester.

Photocatalytic Experiments

Photocatalytic experiments were conducted in bespoke quartz light reactor. Hydrogen production was achieved by dispersing a range of photocatalysts separately in 25 mL mixed solution (MeOH: H_2_O = 1:4). Ascorbic acid (L-AA) was employed as a sacrificial electron donor. Additionally, sodium ascorbic acid (Na-AA) and L-AA were utilized to create a buffer solution [c(A^-^) = 0.1 M, pH = 4] and H_2_PtCl_6_·6H_2_O as platinum precursor (Pt added directly during the photocatalytic reaction, the amount of Pt added was kept constant relative to the reaction volume). A 300W xenon lamp (CEL-HXF300-T3, Au-light) fitted with a cut off filter (λ > 420 nm) served as light source. Prior to the photocatalytic reaction, the reactant solution was subjected to ultrasonication treatment for 20 minutes and bubble with N_2_ for 25 minutes to completely remove the air. The evolved gases were subjected to analysis by gas chromatography (Varian 450-GC, N_2_ carrier) at hourly intervals, with a known concentration of hydrogen employed as the standard gas.

The Apparent Quantum Yield (AQY) was measured using the same method as the PHE by making use of band - pass filter of 420, 450, 500 and 550 nm with the intensities of 100 mW cm^-2^. The irradiation area was controlled as 12.5 cm^2^. Depending on the amount of hydrogen evolution during an average of one-hour photocatalytic reaction, the AQY was calculated based on the equation below:

$$\text{AQY}\text{ = }\frac{\text{N}_{\text{e}}}{\text{N}_{\text{p}}}\text{×100\%}$$

$$\text{=}\frac{\text{2×M×}\text{N}_{\text{A}}}{{\text{E}_{\text{total}}}/{\text{E}_{\text{photon}}}}\text{×100\%}$$

$$\text{=}\frac{\text{2×M×}\text{N}_{\text{A}}}{\text{(S×P×t)}/{\text{(h×}\frac{\text{c}}{\text{λ}}\text{)}}}\text{×100\%}$$

$$\text{=}\frac{\text{2×M×}\text{N}_{\text{A}}\text{×h×c}}{\text{S×P×T×λ}}\text{×100\%}$$

Where, M is the amount of H_2_ (mol), N_A_ is Avogadro constant (6.022×10^23^/mol), h is the Planck constant (6.626×10^-34^ J·s), c is the speed of light (3×10^8^ m/s), S is the irradiation area (cm^2^), P is the intensity of irradiation light (W/cm^2^), t is the photoreaction time (s), λ is the wavelength of the monochromatic light (m).

Electrochemical measurement

Mott Schottky curve (M-S), Electrochemical Impedance Spectroscopy (EIS), LSV curves and Photocurrent–time plot (i-t) were measured using a CHI 760e electrochemical station at a standard three electrode system. The 2 mg X-4 were dispersed in ethanol (0.5 mL) containing 1% nafion (50 μL) by ultrasounding for 10 min. The work electrodes were prepared via drop-casting the 30 μL mixing solution onto the surface of FTO glass substrate electrode used pipette and dried. The Pt plate was employed as the counter electrode and the saturated Ag/AgCl electrode was used as the reference electrode. 0.5 M Na_2_SO_4_ solution was act as an electrolyte. A 300 W xenon lamp equipped with a 420 nm cut-off filter (100 mW cm^-2^) was used as a light source.

Dynamic exfoliation and reversible experiment of I-4

Initial solutions of I-(1-3) and X-4 were prepared at a concentration of 0.3 mg/mL after 1 h ultrasonic treatment, and conduct preliminary screening of their dispersion stability by measuring the zeta potential.

Gradient dilution for particle size and zeta potential testing: prepare an initial solution of I-4 at a concentration of 0.1 mg/mL. Centrifuge for 10 min at 5000 rpm. Take the supernatant and dilute it by 1, 2, 4, and 8 times respectively.

Reversible experiment: add sufficient NaI (solubility 1.84 g/mL) to the I-4 solution (1 mg/mL) to increase the concentration of I^-^ in the solution, and conduct a closed-loop recovery experiment.

**Density Function Theory (DFT) calculation**

Ground-state geometry optimizations were carried out using density functional theory (DFT) with the hybrid meta-GGA exchange-correlation functional M06-2X, as implemented in the Gaussian 16 program package. The def2-SVP basis set was employed for all atoms (C, H, N, O, Cl, Br, I). No symmetry constraints were imposed during optimization, and the convergence criteria for energy, gradient, and displacement were set to the default values in Gaussian 16. All optimized structures were confirmed as local minima by frequency analysis (no imaginary frequencies). Interaction Region Indicator (IRI) analyses were performed using the Multiwfn program based on the electron density and reduced density gradient computed from the optimized wavefunctions. The IRI isosurfaces were visualized and rendered using VMD (Visual Molecular Dynamics) software. All IRI plots were generated with the same color scale and isovalue for consistent comparison across different X‑4 fragments.

**Synthesis of 1, 3, 5-tris (4-formylphenyl) triazine (TFPT)^1^**

**Synthesis of 4', 4''', 4'''''-nitrilotris [(1, 1'-biphenyl) 4-carbaldehyde] (TBPA)^1^**

**Synthesis of N-ethyl-2, 4, 6-trimethylpyridinium bromide (ETMP-Br)^1^**

**Synthesis of N-ethyl-2, 4, 6-trimethylpyridinium bromide (ETMP-I) ^2^**

2,4,6-trimethylpyridine (1.25 mL, 10.0 mmol) and ethyl iodide (3.2 mL, 40.0 mmol) were sequentially dropped into acetonitrile (50 mL) and refluxed overnight at 100 °C. Evaporate the mixed solution under reduced pressure to form yellow solid, which are purified by recrystallization in ethyl acetate to obtain light yellow crystals. Yield: 80.4%. [^1^H NMR (400 MHz, CDCl_3_): 7.55 (s, 2H), 4.75-4.69 (q, 2H), 2.96 (s, 6H), 2.55 (s, 3H), 1.55-1.49 (t, 3H)].

**Synthesis of N-ethyl-2, 4, 6-trimethylpyridinium bromide (ETMP-Cl)**

ETMP-I (500 mg, 1.8 mmol) and AgCl (500 mg, 3.5 mmol) were added to 10 mL H_2_O, sonicated for 15 minutes, and reacted at 60 ℃ in the dark for 12 h. Filter to remove solids, recrystallize the filtrate three times with acetone and evaporate under reduced pressure to obtain white solid. Yield: 84.1%. [^1^H NMR (400 MHz, CDCl_3_): 7.56 (s, 2H), 4.90-4.82 (q, 2H), 2.99 (s, 6H), 2.53 (s, 3H), 1.53-1.48 (t, 3H)].

**Synthesis of N-ethyl-2, 4, 6-trimethylpyridinium bromide (ETMP-Ac)**

ETMP-I (500 mg, 1.8 mmol) and CH_3_COOAg (601.2 mg, 3.6 mmol) were added to 10 mL H_2_O, sonicated for 15 min, and reacted at 60 ℃ in the dark for 12 h. Filter to remove solids, recrystallize the filtrate three times with acetone and evaporate under reduced pressure to obtain purple solid. Yield: 71%. [^1^H NMR (400 MHz, CDCl_3_): 7.49 (s, 2H), 4.85-4.77 (q, 2H), 2.96 (s, 6H), 2.53 (s, 3H), 1.53-1.48 (t, 3H)].

**Synthesis of I-1**

Firstly, dry Pyrex tube was cooled in vacuum and purged with argon for ten minutes. Then ETMP-I (55.5 mg, 0.2mmol), TFPT (78.68 mg, 0.2 mmol), 2 M dimethylamine in anhydrous THF solution (600 μL, 1.2 mmol), 7 mL DMF and 3 mL o-DCB (Extra Dry) were added to Pyrex tube in turn in argon atmosphere. Afterwards, gas exchange through vacuum-argon rapidly and ultrasound for ten minutes. Finally, the Pyrex tube was heated by the metal bath at 180 ℃ for 3d. The obtained precipitate was washed with methanol, tetrahydrofuran, and acetone successively and dried at 80 °C under vacuum overnight to give brown solid. Yield: 88.1%

**Synthesis of I-2**

Firstly, dry Pyrex tube was cooled in vacuum and purged with argon for ten minutes. Then ETMP-I (55.5 mg, 0.2 mmol), TAPB (78.20 mg, 0.2 mmol), 2 M dimethylamine in anhydrous THF solution (600 μL, 1.2 mmol), 7 mL DMF and 3 mL o-DCB (Extra Dry) were added to Pyrex tube in turn in argon atmosphere. Afterwards, gas exchange through vacuum-argon rapidly and ultrasound for ten minutes. Finally, the Pyrex tube was heated by the metal bath at 180 ℃ for 3d. The obtained precipitate was washed with methanol, tetrahydrofuran, and acetone successively and dried at 80 °C under vacuum overnight to give brown solid. Yield: 89.9%

**Synthesis of I-3**

ETMP-I (11.1 mg, 0.04 mmol), TPA (13.2 mg, 0.04 mmol), C_2_H_5_ONa (20.4 mg, 0.3 mmol), 1mL o-DCB and 1mL n-BuOH were loaded into Pyrex tube (o.d. × i.d. = 19 × 16 mm) and sonicated for 10 min. Through three refrigeration cycles at 77 K in liquid nitrogen bath, the tube was sealed under vacuum. Then, the Pyrex tube was heated in thermostatic aluminum block at 120 °C for 3 days. The obtained precipitate was washed with methanol, tetrahydrofuran, and acetone successively and dried at 80 °C under vacuum overnight to give red solid. Yield: 84.39%

**Synthesis of Cl-4**

ETMP-Cl (7.41 mg, 0.04 mmol), TBPA (22.31 mg, 0.04 mmol), C_2_H_5_ONa (20.4 mg, 0.3 mmol), 1mL o-DCB and 1mL n-BuOH were loaded into Pyrex tube (o.d. × i.d. = 19 × 16 mm) and sonicated for 10 minutes. Through three refrigeration cycles at 77 K in liquid nitrogen bath, the tube was sealed under vacuum. Then, the Pyrex tube was heated in a thermostatic aluminum block at 120 °C for 3d. The obtained precipitate was washed with methanol, tetrahydrofuran, and acetone successively and dried at 80 °C under vacuum overnight to give yellow solid. Yield: 86.9%

**Synthesis of Ac-4**

ETMP-Ac (8.33 mg, 0.04 mmol), TBPA (22.31 mg, 0.04 mmol), C_2_H_5_ONa (20.4 mg, 0.3 mmol), 1mL o-DCB and 1mL n-BuOH were loaded into Pyrex tube (o.d. × i.d. = 19 × 16 mm) and sonicated for 10 minutes. Through three refrigeration cycles at 77 K in liquid nitrogen bath, the tube was sealed under vacuum. Then, the Pyrex tube was heated in a thermostatic aluminum block at 120 °C for 3d. The obtained precipitate was washed with methanol, tetrahydrofuran, and acetone successively and dried at 80 °C under vacuum overnight to give brown solid. Yield: 89.06%

**Synthesis of Br-4^1^**

**Synthesis of I-4**

ETMP-I (11.1 mg, 0.04 mmol), TBPA (22.31 mg, 0.04 mmol), C_2_H_5_ONa (20.4 mg, 0.3 mmol), 1mL o-DCB and 1mL n-BuOH were loaded into Pyrex tube (o.d. × i.d. = 19 × 16 mm) and sonicated for 10 minutes. Through three refrigeration cycles at 77 K in liquid nitrogen bath, the tube was sealed under vacuum. Then, the Pyrex tube was heated in thermostatic aluminum block at 150 °C for 3d. The obtained precipitate was washed with methanol, tetrahydrofuran, and acetone successively and dried at 80 °C under vacuum overnight to give red solid. Yield: 87.5%

**Figure. S1** XPS electron binding energy of I_3d_ and Ag_3d_ for (a, b) ETMP-Cl and (c, d) ETMP-Ac.

**Note:** The XPS spectra of ETMP-Cl and ETMP-Ac did not exhibit any photoelectron peaks associated with I or Ag, which suggests complete conversion from ETMP-I to ETMP-Cl and ETMP-Ac.

**Figure. S2** Testing the Cl^-^ and I^-^ mass fractions of ETMP-Cl using ion chromatography.

**Figure. S3** FTIR spectra of X-4 (X=Cl, Ac, Br) and I-(1-3).

**Figure. S4** (a) FTIR spectra and (b) ^13^C CP/MAS NMR of I-4.

**Figure. S5** ^13^C CP/MAS NMR of (a) Cl-4 and (b) Ac-4.

**Figure. S6** ^13^C CP/MAS NMR of (a) I-1, (b) I-2 and (c) I-3.

**Figure. S7** TEM images and corresponding elemental mappings of Cl-4.

**Figure. S8** TEM images and corresponding elemental mappings of Ac-4.

**Figure. S9** TEM images and corresponding elemental mappings of Br-4.

**Figure. S10** TEM images and corresponding elemental mappings of I-4.

Figure. S11 Pawley refinements against the experimental PXRD patterns of (a) Cl-4, (b)Ac-4, (c) I-1 and (d) I-2.

**Figure. S12** N_2_ adsorption/desorption isotherm and pore size distribution of (a, b) I-1, (c, d) I-2.

**Figure. S13** N_2_ adsorption/desorption isotherm of (a) I-3 and (b) I-4.

**Figure. S14** N_2_ adsorption/desorption isotherm and pore size distribution of (a, b) Cl-4 and (c, d) Ac-4.

**Figure. S15** Thermal gravimetric analysis (TGA) profiles of X-4.

**Figure. S16** Tauc plot analysis of X-4.

**Figure. S17** The Mott-Schottky plots of (a) Cl-4 (b) Ac-4 (c) Br-4 (d) I-4.

**Figure. S18** Temperature-dependent PL spectra and integrated PL emission intensity as a function of temperature from 80 to 300 K of as-prepared (a, b) Cl-4, (c, d) Ac-4, (e, f) Br-4, (g, h) I-4.

**Figure. S19** Photoluminescence lifetime spectra of X-4.

**Figure. S20** EIS Nyquist plots under light-dark conditions for (a) Cl-4, (b)Ac-4, (c)Br-4, (d) I-4^3^.

**Table. S1** Resistance value (R_s_ and R_c_) of X-4.

| Sample | | R_s_ (ohm) | R_c1_ (ohm) | R_c2_ (ohm) |
| --- | --- | --- | --- | --- |
| Cl-4 | **light off** | 24.19 | 1.34*10^5^ | 2.27*10^6^ |
|  | **light on** | 24.42 | 1.12*10^5^ | 2.39*10^6^ |
| Ac-4 | **light off** | 25.89 | 1.46*10^5^ | 7.63*10^6^ |
|  | **light on** | 26.11 | 5.72*10^6^ | 1.18*10^5^ |
| Br-4 | **light off** | 21.69 | 6.99*10^4^ | 4.22*10^6^ |
|  | **light on** | 21.28 | 6.49*10^4^ | 2.10*10^6^ |
| I-4 | **light off** | 25.25 | 5.03*10^4^ | 3.16*10^6^ |
|  | **light on** | 25.58 | 3.94*10^4^ | 1.28*10^6^ |

**Figure. S21** Steady-state PL spectroscopy of X-4.

**Figure. S22** LSV curves of (a) X-4 and light-dark contrast for (b) Cl-4, (c)Ac-4, (d)Br-4, (e) I-4.

**Figure. S23** The optimal loading amount of Pt for I-4 with different qualities.

**Figure. S24** The PHE performance of 0.5 mg I-4 at 25 ℃ and 35 ℃, respectively.

**Figure. S25** Hydrogen evolution efficiency of 0.5 mg I-4 under dark and catalyst free conditions while keeping other conditions constant.

**Figure. S26** Time course of PHE under visible light irradiation using 2 mg I-4 for 15 h.

**Figure. S27** (a)FTIR and (b) PXRD spectra of I-4 before (black) and after (red) photocatalysis. The characterization ware performed after 15 h H_2_ evolution experiment.

**Figure. S28** TEM images and corresponding elemental mappings of I-4 after photocatalysis. The characterization ware performed after 15 h H_2_ evolution experiment.

**Figure. S29** AFM images of I-4 after photocatalysis. The characterization ware performed after 15 h H_2_ evolution experiment.

**Figure. S30** Wavelength-dependent AQY of photocatalytic H_2_ evolution for I-4.

**Figure. S31** Zeta potential by diluting the concentration of the original solution for (a) Cl-4, (b) Ac-4 and (c) Br-4.

**Figure. S32** Determination of the normal distribution curve for I-4 particle size by diluting the concentration of the I-4 stock solution.

**Note:** Using water as the solvent, the original solution of I-4 at a concentration of 0.1 mg/mL, was subjected to1 h ultrasonic treatment and subsequently diluted 1, 2, 4, and 8 times, respectively, to test the particle size and zeta potential.

**Figure. S33** Particle size by diluting the concentration of the original solution for Br-4.

**Note:** Using water as the solvent, the original solution of Br-4 at a concentration of 0.1mg/mL, was subjected to1 h ultrasonic treatment and subsequently diluted 1, 2, 4, and 8 times, respectively, to test the particle size.

**Figure. S34** AFM image of (a) Cl-4, (b) Br-4 (c) Ac-4 and (d) multilayer stacked Ac-4.

**Note:** AFM images of Cl-4, Br-4, and Ac-4 were tested under low concentration conditions.

**Figure. S35** HR TEM images of Cl-4 at (a) high concentrations and (b) low concentrations.

**Figure. S36** HR TEM images of Ac-4 at (a) high concentrations and (b) low concentrations.

**Figure. S37** HR TEM images of Br-4 at low concentration.

**Note:** The detailed information of HR TEM for Br-4 can be found in our previous work^1^.

**Figure. S38** SEM images of I-4 at (a) high concentrations and (b) low concentrations.

**Figure. S39** HRTEM images of I-4 at (a) high concentrations and (b) low concentrations.

**Figure. S40** Comparative photographs of the sedimentation of X-4 and I-(1-3) at the same concentration.

**Figure. S41** Contact angle images at different time intervals of X-4.

**Figure. S42** The dispersion status of I-4 with different qualities in the catalytic system.

**Figure. S43** The aggregation situation of I-4 colloid over time subsequent to the addition of NaI.

**Figure. S44** The hydrogen evolution performance of I-4 after closed-loop recovery.

**Table. S2** The orbital distribution of X-4.

|  | HOMO | HOMO-3 | LUMO |
| --- | --- | --- | --- |
| Cl-4 |  |  |  |
| Ac-4 |  | -- |  |
| Br-4 |  |  |  |
| I-4 |  |  |  |

**Note:** HOMO, HOMO-1, and HOMO-2 are the outermost three p orbitals of Cl^-^, Br^-^, and I^-^, respectively. Their energies are in close proximity, therefore, only the distribution of HOMO orbitals is shown. Unlike Cl-4, B-4r, and I-4, the HOMO distribution of Ac-4 is observed to be in proximity to triphenylamine, rather than on Ac^-^.

|  | VBM (eV) | CBM  (eV) | HOMO (eV) | LUMO (eV) | Eg_opt_ (eV) | Eg_elec_ (eV) | HOMO-3 (eV) | Eg_elec_^a^ (eV) |
| --- | --- | --- | --- | --- | --- | --- | --- | --- |
| Cl-4 | **2.11** | **-0.51** | **-5.80** | **-2.45** | **2.62** | **3.35** | **-6.05** | **3.6** |
| Ac-4 | **2.02** | **-0.47** | **-6.10** | **-2.42** | **2.49** | **3.68** | **--** | **--** |
| Br-4 | **1.14** | **-0.84** | **-5.44** | **-2.53** | **1.98** | **2.91** | **-6.06** | **3.53** |
| I-4 | **0.80** | **-1.12** | **-5.13** | **-2.62** | **1.92** | **2.51** | **-6.08** | **3.46** |

**Table. S3** Electronic properties with X-4.

*Eg_elec_^a^: The band gap between HOMO-3 and LUMO.*

**Figure. S45** IRI analysis of local fragment for (a) Cl-4, (b) Ac-4, (c) Br-4 and (d) I-4 showing the non-covalent interactions between the cationic skeleton and counter anion. The colour scale is in the range of -0.02 < ρ < -0.01 a.u. Blue, green and red indicate regions of bond forming and notable interaction, moderate interaction, weak interaction and repulsion, respectively.

**Table. S4** The distance between X and H on the skeleton in X-4.

|  | d_X-H1_ (Å) | d_X-H2_ (Å) | d_X-H3_ (Å) | d_avg_ (Å) |
| --- | --- | --- | --- | --- |
| Cl-4 | 2.27 | 2.27 | 2.37 | 2.30 |
| Ac-4 | 2.09 | 1.94 | 2.02 | 2.02 |
| Br-4 | 2.46 | 2.55 | 2.57 | 2.53 |
| I-4 | 2.69 | 2.87 | 2.82 | 2.79 |

**References**

1.J. Yang, X. Zhang, W. Si*, et al.*,"Alkene-Bridged Ionic Covalent Organic Nanosheets (iCONs) Based on D-π-A for Photocatalytic Hydrogen Evolution," *ACS Catalysis* **14** (2024): 2022-2030. <https://doi.org/10.1021/acscatal.3c030341>.

2.F. Meng, S. Bi, Z. Sun*, et al.*,"Synthesis of Ionic Vinylene-Linked Covalent Organic Frameworks through Quaternization-Activated Knoevenagel Condensation," *Angewandte Chemie International Edition* **60** (2021): 13614-13620. <https://doi.org/10.1002/anie.2021043752>.

3.Y. Liu and Y. Xu,"Porous membrane host-derived in-situ polymer electrolytes with double-stabilized electrode interface enable long cycling lithium metal batteries," *Chemical Engineering Journal* **433** (2022): 134471. <https://www.sciencedirect.com/science/article/pii/S13858947210604113>.
